# Genetic polymorphism of CCR5 (59029A/G) and CCR2 (-64I A/G) promoters in HIV infected patients

**DOI:** 10.12669/pjms.41.9.12174

**Published:** 2025-09

**Authors:** Aafshar Khalid, Romeeza Tahir, Shah Jahan, Hasnain Javed

**Affiliations:** 1 Aafshar Khalid, M.Phil. Department of Immunology, University of Health Sciences, Lahore, Pakistan; 2 Romeeza Tahir, Ph.D. Department of Immunology, University of Health Sciences, Lahore, Pakistan; 3 Shah Jahan, Ph.D. Institute of Allied Health Sciences, University of Health Sciences, Lahore, Pakistan; 4 Hasnain Javed, Ph.D. Provincial Public Health Reference Lab, Punjab AIDS Control Program Lahore, Lahore, Pakistan

**Keywords:** AIDS, HIV, CCR2(-64I), CCR5(59029A/G), CD4^+^T cell

## Abstract

**Background and Objective:**

Human immunodeficiency virus (HIV) is responsible for acquired immunodeficiency syndrome (AIDS), which is the final stage of infection by HIV. This study investigated the role of the CCR5-59029A/G and CCR2-64IA/G polymorphisms in HIV susceptibility in a local Pakistani population. These two chemokine receptors facilitate HIV entry into host cells, making them key genetic markers for understanding the disease progression.

**Methodology:**

This case-control study was conducted at the Laboratory of Immunology, University of Health Sciences (UHS), Lahore from July 2022 till March 2023. Blood specimens were collected of 60 newly diagnosed HIV-infected patients from Provincial Public Health Reference Lab Lahore and 60 healthy controls. PCR was followed by restriction fragment length polymorphism (RFLP) analysis with gene-specific primers and enzymes (Bsp1286I and FokI).

**Results:**

The CCR5-59029A/G polymorphism was wild-type across all participants, with no mutations detected. For the CCR2-64IA/G mutation, a minor frequency of 3.34% was observed in healthy controls, whereas no mutations were found in HIV-positive individuals. These results suggest that neither the CCR5-59029A/G nor CCR2-64IA/G polymorphisms are linked to HIV susceptibility in this population, with both groups showing a high prevalence of the wild-type forms.

**Conclusion:**

CCR5-59029A/G and CCR2-64IA/G SNPs did not appear to influence susceptibility in this local Pakistani population. This work underscores the regional variability of genetic factors in HIV research and highlights the need for further investigation of other possible contributors to HIV resistance.

## INTRODUCTION

AIDS has a significant impact on public health and is the last stage of HIV infection. During HIV infection, targeting CD4^+^ T cells leads to their destruction.^1^ The body’s defense against HIV relies on the activation of both the innate and adaptive immune mechanisms. The host response can only slow viral multiplication and briefly postpone the effects of infection, despite efforts to completely remove the virus. HIV uses several immune evasive strategies throughout the infection, including hiding in host cells to ensure their survival and replication. The extended incubation and infectious periods of HIV infection are also widely recognized. Without antiretroviral therapy, the average time HIV infection takes to progress to AIDS is approximately ten years.^2^

The average time that people live after contracting the disease is approximately ten months. Since the discovery of HIV in the 1980s, the mortality rate in underdeveloped nations has remained high. HIV remains a serious threat to global health, infecting millions of people annually 3 Pakistan is highly susceptible to HIV/AIDS epidemics. HIV prevalence among people who inject drugs in Pakistan was thought to be around 20% in the early 2000s, but current observed data from 2016/17 showed that HIV prevalence has increased to 38%. Several socioeconomic factors promote viral transmission, such as a high rate of unemployment, lack of education, and poverty.^4^,^5^

The dynamic interaction between HIV and the chemokine system has profoundly enhanced our insight into AIDS pathogenesis, paving the way for novel approaches to prevention and treatment strategies.^6^ During the infection of HIV-1, CD4 and co-receptors on T cells enable viral entry, with chemokine receptors playing a pivotal role. Specifically, CCR5 and CXCR4 are considered the primary entry points.^7^ CCR5 is a member of the large chemokine receptor group located on the lymphocyte surface, as well as on other cells, including monocytes, macrophages, immature DC, Th1 cells, astrocytes, microglia, epithelium, endothelium, vascular smooth muscle, and fibroblasts. CCR5 is one of the genes that has been identified as the key co-receptor in HIV infection.^8^

The CCR5 gene has four exons, two introns, and two promoters: a relatively weak upstream promoter and a strong downstream promoter. SNP located in the promoter region or coding area of CCR5 leads to alterations or total suppression of CCR5 receptor protein. Various CCR5 gene polymorphisms, including CCR5-m303, CCR5-59653T, and the CCR5 promoter 59029A/G, have been linked to HIV susceptibility. The CCR5 59029A/G transition, located at base pair 59029 in the CCR5 promoter, shows common allelic frequencies, with 59029-A ranging from 43-68% depending on ethnicity.^9^ However, the distribution and impact of CCR5 promoter 59029A/G on HIV-1 infection in Pakistan remain unexamined.^10^

CCR2 acts as the main receptor for monocyte chemoattractant protein-1 (MCP1) and is found in a variety of immune cells, such as macrophages, activated T cells, and monocytes.^11^ Several allelic SNPs in CCR2 have been investigated for disease proneness or severity.^12^ The most extensively investigated CCR2 mutation is GT to AT. This polymorphic variation occurs in the coding region at nucleotide position 190; as a result, valine is replaced with isoleucine at the 64^th^ position of the amino acid. This variant is denoted as CCR2-V64I and affects the expressed protein.^13^ This mutation has been linked to a large delay in the development of AIDS in some cohorts, although it shows no protective effect against HIV infection. The prevalence of these genetic variants, particularly among high-risk populations that are HIV-1 seropositive or seronegative, remains unexplored in Pakistan. Gaining insight into how these genetic variants influence susceptibility to HIV-1 infection within the country is crucial for advancing our understanding of and response to the epidemic.^10^

## METHODOLOGY

This case-control study was conducted from July 2022 till March 2023 in the department of Immunology, UHS Lahore. The sample size was calculated by keeping the power of the study equal to 90% and the level of significance equal to 5%.^14^ Sixty patients from the Punjab AIDS Control Program and 60 age-matched healthy controls of both genders were recruited. All HIV-infected patients were newly diagnosed adults. HIV-infected patients on antiretroviral therapy and those less than 18 years of age were excluded from this study. Informed consent was obtained from all study participants.

### Ethical Approval:

This study was approved by the Ethics Committee (UHS/REG-22/ERC/AK) and the Advanced Studies and Research Board (UHS/Education/126-22/8740; Dated: August 26, 2022) of UHS.

### Genotyping of CCR5(59029A/G) and CCR2(-64I) gene polymorphism:

Genomic DNA was extracted from whole blood using the phenol-chloroform method 15 in a Thermo Scientific Extraction Cabinet. DNA quality and quantity were assessed using a NanoDrop spectrophotometer (Thermo Scientific, Waltham, MULTISKAN, USA) and stored at -20°C.

The promoter (59029A/G) polymorphism of the CCR5 gene was analyzed using the PCR-RFLP (Polymerase Chain Reaction- Restriction Fragment Length Polymorphism) method.^10^ The CCR5 promoter region (59029A/G) was amplified using specific primers (Table-I) and a standard PCR protocol.^16^ The amplified products were separated on a 3% agarose gel, and the resulting bands were visualized using a UV transilluminator. Restriction digestion for CCR5(59029A/G) polymorphism was performed using the Bsp1286I enzyme (10U/ μl)^10^ in a standard reaction mixture. The gene polymorphism of CCR2-64I was analyzed using PCR followed by RFLP, utilizing established protocols.^8^ CCR2 was amplified using the primers listed in Table-I. The reaction mixture (up to 25 μL) was prepared similarly to the CCR5 (59029A/G) analysis. For RFLP analysis, 4 μL of the PCR product was incubated in a reaction mixture of 15 μL containing 4U of Fast Digest FokI enzyme 17 at 37°C for 15 min. The digested products were resolved on 4% agarose gel.

**Table I:** Primers used in the study.

Genetic variants	Primer’s sequence	Size of Amplicon	Digestion Enzyme	Allele	Size of Fragments
CCR5(59029A/G)	TGGGGTGGGATAGGGGATAC TGTATTGAAGGCGAAAAGAATCAG	498bp	Bsp1286I	A	453bpb
				G	45bp
CCR2(-64I)	GGATTGAACAAGGACGCATTTCCCC TTGCACATTGCATTCCCA	380bp	FokI	I	215bp
				V	167bp

### Statistical Analysis:

This study assessed polymorphisms for deviation from Hardy-Weinberg equilibrium, employing the chi-square test to assess differences between observed and expected genotype frequencies. The frequency of CCR5 promoter 59029 A/G and CCR2-64I polymorphism and alleles were compared across different groups using the chi-square test. A p-value of ≤ 0.05 was deemed statistically significant.

## RESULTS

**Fig.1:**
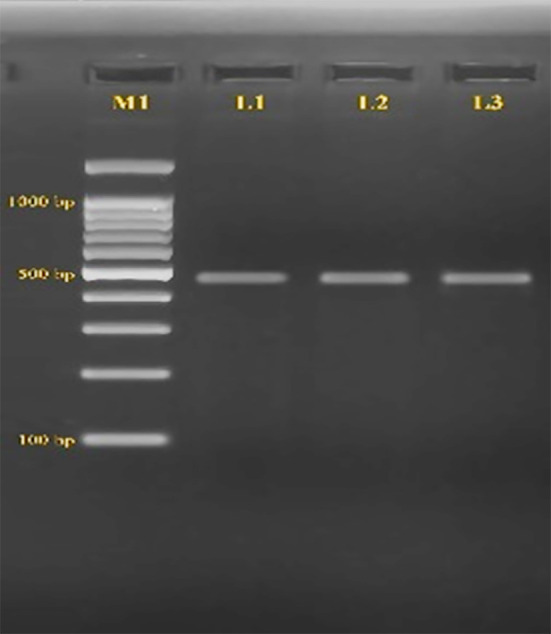
Restriction fragment length polymorphism analysis of CCR5-59029 A/G polymorphism. M1: 100 bp DNA ladder; M2: 50 bp DNA ladder; L1-L2: wild-type CCR5-59029 A/G genotype showing a single 498 bp fragment; L3: undigested PCR product (498 bp).

The baseline characteristics of the study participants are summarized in Table-II. The CCR5-promoter (59029 A/G) was amplified and revealed a band of 498bp. After amplification, RFLP was performed on CCR5(59029A/G) using Bsp1286I. No genetic polymorphism was found, either homozygous or heterozygous, in the HIV-infected and healthy groups in this study. All 120 participants had wild-type genotypes, as shown in Fig.1.

**Table II:** Demographic distribution of study participants by age and gender.

Variables		Newly diagnosed HIV patients (n=60)	Healthy controls (n=60)
Age Median (Years)		45[34-66]	37[34-56]
Gender	Male	48(80%)	34(56.7%)
	Female	12(20%)	26(43.3%)

**Fig.2:**
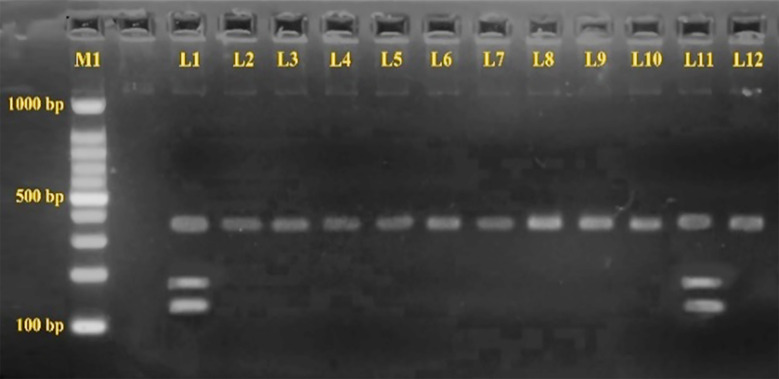
Restriction fragment length polymorphism analysis of CCR2(-64I A/G) polymorphism. M1: 100bp ladder, L2-L10, L12: wild-type fragment of CCR2(-64I A/G) (380bp fragment) L1, L11 heterozygous A/G genotype (125bp + 165bp fragment).

The CCR2(-64I A/G) gene was amplified by PCR and revealed a band of 380 bp. The gene was then analyzed for mutation by RFLP. Amplified DNA samples were genotyped for the polymorphism of CCR2(-64I A/G). The calculated frequency of the CCR2(-64I A/G) gene variant was 3.34%, and the variant was only observed in control subjects, while the frequency of wild-type CCR2(-64I A/G) was 96.6%. No mutations were observed in the HIV-infected population (Fig.2).

In the local population, the frequency of the G allele was 98% and that of the A allele was 2%. In the HIV-infected group, the G allele frequency was 100% and the A allele was absent. Overall, the frequency of the A allele in the study cohort was 2% (Table III).

**Table-III:** Distribution of CCR2-64I Gene polymorphism.

CCR2(-64I)	Newly diagnosed HIV patients	Healthy controls	OR (95% CI)	p-value
G/G^α^	60 (100%)	58 (96.7%)	-0.0121-0.0788	0.496
A/G^β^	0 (0%)	2 (3.3%)	NA*	NA*
A/A^γ^	Not detected	Not detected	-	-

α: Wild-type homozygotes β: Mutant heterozygous γ: Mutant homozygous;

* NA: Not Applicable [There were 0 patients with a specific allele, so the OR and p-value cannot be calculated (mathematical limitation]

## DISCUSSION

The first case of HIV infection in Pakistan was reported in 1987. According to reports from the World Health Organization, Pakistan is the second-largest HIV-positive nation in Asia, with an estimated 200,000 cases 4 Numerous investigations have examined the relationship between host vulnerability to HIV-1 infection and polymorphisms in chemokines and their receptor genes; however, the results are controversial. CCR5 (59029A/G) and CCR2 (-64I) alleles confer a protective effect against the progression of HIV-1 infection. For macrophage-tropic HIV strains, the primary coreceptor is CCR5. When CCR5 is not present on the surface, it prevents HIV from entering host cells.^5^ CCR2 plays a vital role as a co-receptor that facilitates the entry of HIV-1 into CD4^+^ host cells. CCR2 alleles contribute to illness progression, but not disease onset suggesting that CCR2 alleles could influence the rate of HIV disease progression after infection but do not affect the initial risk of acquiring the virus.^9^

To the best of our knowledge, there is currently a lack of published data on CCR5(59029) and CCR2-64I in HIV from Pakistan. The absence of such data highlights the novelty and significance of this study as it provides new insight. Discovering the CCR5 promoter 59029A/G and CCR2-64I allelic variants, as well as their distribution, may aid in understanding disease burden and progress, which could aid in therapeutic decision-making. Polymorphisms in CCR5 and CCR2 have been found to influence an individual’s vulnerability to HIV-1 infection.^7^

In our local population, no SNP were homozygous or heterozygous. No allelic differences were observed between infected and healthy subjects. However, in Cameroon, the prevalence of this SNP was 50%. The SNP of CCR5 promoter 59029 A/G was identified in approximately 50% (49.72%) of the healthy population. However, there was no significant difference between the healthy and infected populations. Notably, the homozygous G/G genotype appears to be more prevalent among HIV-positive status.^10^ As shown in the present study; it may be rare in a local cohort (0% of our study population). These findings are consistent with those reported by Li *et al*,^18^ Ge Y *et al*,^19^ and Sriwanthana *et al.^20^* Previous studies found no effect on the sensitivity of the host to infection caused by HIV-1. Several studies have indicated that these SNPs may impact disease advancement or AIDS onset, as reported by Anzala *et al,^21^* Claireaux *et al*,^22^ and Winkler *et al*.^23^

The CCR2 allele (-64I) has been linked to slow development of AIDS. Several recent investigations conducted in countries worldwide have revealed that the CCR2 allele (-64I) is widely dispersed.^7^,^24^,^25^ Approximately 13% of the CCR2-64I allele was found in the healthy population of South Africa.^21^ In the Kenyan population, it ranged from 21% to 23%; in Cameroon, 7.1% 10 and in the Turkish population, it was 15.6% -19.3% which was comparable to the results observed in North America and Western Europe, respectively.^7^ The present study, conducted in Pakistan, showed that CCR2-64I allelic frequency in HIV-positive individuals was found to be almost similar to that in the local healthy group. In this study of HIV patients and healthy controls in Pakistan, the CCR2-64I polymorphism was found in 3.3% of the healthy control group. This study found a statistically insignificant difference based on studies conducted by Köksal *et al*.^7^ and Nkenfou *et al*.^10^ Genotype distribution of both gene variants, CCR2-64I and CCR5(59029A/G) were in equilibrium as anticipated by the Hardy-Weinberg equation(p>0.5), indicating that there was an ongoing selection for or against this allele in the study population which is in corroborated with the study of Winkler *et al*.^23^

The findings of this study support the need to search for variants in the human genome that influence illness development in general as well as their impact on CD4^+^T cell depletion. Even if the biological basis for the novel polymorphism and association is not clear, new avenues of experimental investigation may be developed. While previous studies from other geographic regions have demonstrated that certain variants of CCR5 and CCR2 can alter the risk of HIV acquisition and progression, our findings indicate that these specific polymorphisms are predominantly wild type in this cohort. Strengths of the study include the use of a well-defined case-control design, confirmed newly diagnosed HIV cases to minimize the effect of treatment-related viral or immunological changes, and validated molecular methods for genotyping. The recruitment of participants from a provincial reference laboratory also ensured accurate diagnosis and minimized misclassification bias.

### Limitations and future directions:

While the sample size was adequate for detecting common alleles, the rarity of these mutations in our population limits the ability to assess their effect on HIV progression. Future studies should expand sample sizes and investigate a broader panel of genetic markers involved in HIV entry and immune response, including the CCR5-Δ32 mutation, CXCR4-related variants, and HLA alleles. Additionally, integrating genetic data with virological, and immunological parameters could help identify multifactorial determinants of HIV susceptibility and resistance in the Pakistani cohort.

## CONCLUSION

It was concluded that the distribution of two HIV-related gene isoforms, CCR5 (59029A/G) and CCR2-64I, among individuals infected with HIV did not differ from that of healthy individuals. No difference was observed in allelic frequency, suggesting that these SNPs were not associated with disease susceptibility. There was no statistically significant evidence to support the hypothesized relationship between genetic polymorphisms of both CCR5 (59029A/G) and CCR2- 64I in HIV-infected and healthy subjects.
